# Development and external validation of a risk prediction model for hypertension among individuals with established hypothyroidism

**DOI:** 10.3389/fendo.2026.1945343

**Published:** 2026-09-03

**Authors:** Lan Lin, Liqing Chen, Kailing Yu, Qianwen Li, Junqian Huang, Ling Li, Yizhen Chen, Xiaoling Ke, Junping Wen, Jianbin You

**Affiliations:** 1 Center for Information Management, Fuzhou University Affiliated Provincial Hospital, Fuzhou, China; 2 Fujian Provincial Key Laboratory of Medical Big Data Engineering, Fuzhou, China; 3 Department of Clinical Laboratory, Fuzhou University Affiliated Provincial Hospital, Fuzhou, China; 4 Shengli Clinical Medical College of Fujian Medical University, Fujian Provincial Hospital, Fujian Medical University, Fuzhou, China; 5 Department of Endocrinology, Fuzhou University Affiliated Provincial Hospital, Fuzhou, China

**Keywords:** cardiovascular risk, hypertension, hypothyroidism, precision endocrinology, prediction model, risk stratification

## Abstract

**Background:**

Hypothyroidism represents a multisystem endocrine disorder involving cardiovascular, renal, and metabolic alterations beyond thyroid hormone deficiency. However, validated tools for hypertension (HTN) risk stratification specifically designed for patients with hypothyroidism remain lacking. This study aimed to develop and externally validate a hypothyroidism-specific HTN prediction model incorporating routinely available clinical variables.

**Methods:**

This retrospective multicohort study included patients with hypothyroidism from a hospital-based cohort and an independent external population-based cohort. Eligible participants were identified according to predefined diagnostic criteria, and individuals with missing key clinical variables or incomplete outcome information were excluded. Candidate predictors were selected using regression-based approaches, and a multivariable prediction model was developed in the derivation cohort and subsequently evaluated in internal and external validation cohorts. An interpretable HTN risk profile was developed and evaluated using internal validation, external validation, and subgroup analyses.

**Results:**

Age, serum creatinine (SCr), anion gap (AG), and diabetes mellitus (DM) constituted a clinically interpretable risk profile reflecting vascular aging, renal vulnerability, and metabolic dysregulation. The model demonstrated robust discrimination, good calibration, and favorable clinical utility across training, testing, and external validation cohorts.

**Conclusions:**

A disease specific HTN risk profile was developed and externally validated in patients with hypothyroidism. This model provides a practical approach for individualized cardiovascular risk stratification and supports the application of precision endocrine medicine.

## Introduction

Hypothyroidism is one of the most prevalent endocrine disorders worldwide. The clinical consequences of hypothyroidism extend beyond isolated thyroid hormone deficiency and involve complex alterations across cardiovascular, renal, and metabolic systems (1, 2). Thyroid hormone deficiency may increase peripheral vascular resistance, impair endothelial function, alter renal physiology, and disrupt metabolic homeostasis, thereby contributing to hypertension (HTN) and cardiovascular risk (3). Therefore, HTN represents an important cardiovascular complication among patients with hypothyroidism. Hypothyroidism is increasingly recognized not only as a state of thyroid hormone deficiency but also as a clinically relevant multisystem vulnerability phenotype (4). Understanding this multisystem vulnerability may provide new insights into cardiovascular risk susceptibility among patients with endocrine disorders.

Despite the established association between hypothyroidism and increased HTN risk, current clinical risk assessment strategies still largely rely on conventional cardiovascular prediction models developed in general populations (5, 6). However, these models may not fully capture the endocrine metabolic characteristics and multisystem alterations associated with hypothyroidism. Unlike models based on individual risk factors, cardiovascular susceptibility in hypothyroidism may involve multiple physiological domains, including vascular aging, renal vulnerability, metabolic imbalance, and impaired glucose regulation (7, 8). Risk stratification approaches specifically designed to capture these disease-related physiological vulnerabilities are needed to improve the early identification of individuals at high risk of HTN. Whether existing cardiovascular risk prediction approaches can accurately stratify HTN susceptibility among patients with hypothyroidism remains unclear. Moreover, disease-specific risk prediction models incorporating endocrine-related metabolic abnormalities for this population remain unavailable.

Precision endocrine medicine emphasizes the integration of multidimensional clinical information to characterize disease heterogeneity and individual variations in risk profiles. Conventional clinical indicators not only provide predictive value but may also serve as surrogate markers of underlying physiological abnormalities. Among these indicators, age may reflect vascular aging, whereas serum creatinine (SCr) represents renal function and renal vulnerability (9, 10). The anion gap (AG) may indicate alterations in metabolic homeostasis (11). Diabetes mellitus (DM) reflects persistent glycemic dysregulation and related cardiovascular risk (12). Together, these variables may form an integrated and clinically interpretable signature for identifying cardiovascular susceptibility among patients with hypothyroidism. Therefore, integrating these routinely available indicators may capture the multidimensional physiological vulnerability associated with HTN development in hypothyroidism and provide a clinically applicable approach for individualized risk assessment.

Against this background, this study aimed to develop and externally validate a hypothyroidism-specific hypertension risk prediction model using routinely available clinical variables and to assess its predictive performance and clinical utility across multiple cohorts. The developed model may facilitate precision cardiovascular risk stratification and individualized risk assessment among patients with hypothyroidism.

## Methods

### Data source

Clinical data from patients with hypothyroidism who visited the Affiliated Provincial Hospital of Fuzhou University between January 2016 and April 2026 were retrospectively collected, and an internal cohort (n = 2754) was established for model development. This study period was selected because electronic medical records and laboratory information systems were consistently available throughout this interval, enabling standardized extraction of demographic characteristics, comorbidities, and laboratory measurements. The study period ended in April 2026 based on the most recent database update. An independent external validation cohort (n = 1191) of patients with diagnosed hypothyroidism from the MIMIC-IV database was used to assess model generalizability. This cohort served as an independent population for evaluating model transportability rather than as an external control group. Predictor definitions, outcome ascertainment criteria, and data preprocessing procedures were harmonized across the internal and external cohorts to reduce potential selection bias and data heterogeneity. The established model was directly applied to the external cohort without recalibration or retraining. The sample size was determined by the availability of eligible patients and the number of observed outcome events. The number of hypertension events was sufficient to meet the recommended events-per-variable (EPV) threshold for prediction modeling. In the internal cohort, 1,283 hypertension events were available for the four predictors included in the final model, resulting in an EPV of approximately 321. Similarly, the external validation cohort included 815 hypertension events, corresponding to an EPV of approximately 204.

All participants included in this study were patients with established hypothyroidism. The exclusion criteria were as follows: (1) patients younger than 18 years were excluded because pediatric hypertension has different diagnostic criteria, developmental characteristics, and pathophysiological mechanisms compared with adult hypertension; (2) missing data for any key variables, including age, gender, HTN, DM, or hypothyroidism. The diagnoses of hypothyroidism, HTN and DM were confirmed through manual review of the corresponding ICD 9 and ICD 10 diagnostic codes (**Supplementary Table 1**). Because the available datasets did not consistently distinguish between type 1 and type 2 diabetes, DM was analyzed as a binary comorbidity variable. Based on HTN status, the internal cohort was classified into the non-HTN group (n = 1471) and the HTN group (n = 1283), while the external cohort was classified into the non-HTN group (n = 376) and the HTN group (n = 815). This study was conducted in accordance with the Declaration of Helsinki and was approved by the Ethics Committee of the Fuzhou University Affiliated Provincial Hospital (Approval No. K2026-07-035). Because this was a retrospective study and all data were anonymized, the requirement for informed consent was waived.

### Data analysis and processing

We collected common clinical variables from both the internal and external datasets, including demographic characteristics, comorbidities, and laboratory measurements. The proportion of missing data was assessed for all variables included in the analysis, and variables with missing rates exceeding 10% were excluded. Ultimately, 25 variables were retained for subsequent analysis, including: age, gender, DM, white blood cell (WBC),red blood cell (RBC), hemoglobin (Hb), hematocrit (Hct), mean corpuscular volume (MCV), mean corpuscular hemoglobin content (MCH), mean corpuscular hemoglobin concentration (MCHC), red cell distribution width (RDW), platelet (PLT), albumin (ALB), total bilirubin (TBil), alanine aminotransferase (ALT), aspartate aminotransferase (AST), alkaline phosphatase (ALP), triglycerides (TG), blood urea nitrogen (BUN), serum creatinine (SCr),potassium (K), sodium (Na), calcium (Ca), magnesium (Mg), anion gap (AG). Missing values were imputed using the MissForest algorithm, a nonparametric random forest-based imputation method that can handle mixed-type variables and nonlinear relationships (13). MissForest has been increasingly applied to biomedical and clinical datasets because of its ability to preserve complex variable structures and enhance predictive modeling performance in the presence of missing data (14).

### Variables selection

The internal dataset was randomly divided into a training set and a testing set at a ratio of 7:3. A 7:3 split was selected because it provides a balance between sufficient sample size for model development and adequate independent data for performance evaluation. This split strategy has been widely adopted in clinical prediction modeling studies and provides reliable internal validation while maintaining adequate events for model training (15). No statistically significant differences were observed in baseline characteristics between the two sets (**Supplementary Table 2**). Least absolute shrinkage and selection operator (LASSO) regression was used for preliminary variable selection. LASSO achieves variable selection by imposing an L1 regularization penalty on the regression coefficients, shrinking the coefficients of less informative variables to zero and thereby excluding them from the model. Compared with traditional stepwise selection approaches, LASSO can perform variable selection and coefficient shrinkage simultaneously, which may reduce overfitting and improve model stability when multiple candidate predictors are considered (16). Subsequently, univariate and multivariate logistic regression analyses were performed to identify the optimal predictors. This approach helps control bias introduced by potential confounding factors while improving the predictive performance and interpretability of the model. The variance inflation factors (VIFs) of the variables selected by LASSO were calculated to assess potential multicollinearity before subsequent regression analyses (17). Variables with VIF values > 5 were considered to exhibit substantial multicollinearity and were excluded from subsequent analyses. After this step, the remaining variables were preliminarily evaluated using univariate and multivariate logistic regression analyses (18). Interaction terms between major predictors were examined, and no statistically significant interactions were observed. The independent predictors identified from the multivariate logistic regression model were used to construct the nomogram.

### Construction and validation of a nomogram for predicting the risk of HTN in patients with hypothyroidism

Based on the training set, the optimal variables were used to develop a nomogram model, which was subsequently validated in the test set and an external validation dataset. Nomograms are widely used clinical prediction tools that integrate multiple predictors to provide individualized risk estimation and require assessment of discrimination, calibration, and clinical utility (19). The area under the receiver operating characteristic curve (AUC), sensitivity, and specificity were used to evaluate the performance of the nomogram model. The calibration curve was used to assess the agreement between the predicted probabilities and the observed outcomes (20). Decision curve analysis (DCA) was used to quantify the net benefit of the model across different threshold probabilities and to assess its clinical utility (21).

A quantitative scoring system was established based on the regression coefficients from the multivariable logistic regression model to enhance the clinical interpretability of the nomogram. Each predictor was assigned a weighted score according to its contribution to the predicted risk. The total score was calculated by summing the scores of all predictors and was subsequently converted into an estimated probability of hypertension. According to the distribution of predicted probabilities and predefined clinically relevant thresholds, patients were classified into three risk groups: low risk (predicted probability < 0.5), moderate risk (0.5 - 0.7), and high risk (> 0.7). This risk stratification method may facilitate individualized assessment of hypertension risk among patients with hypothyroidism.

### Subgroup analyses

Subgroup analyses were performed by age and gender to assess the robustness of the predictive model across different demographic subgroups. Based on the age distribution of hypertensive patients in our cohort and commonly used definitions of older adults in epidemiological studies, participants were categorized into two clinically meaningful age groups: younger than 60 years and 60 years or older (22). Participants were also stratified by gender into male and female groups. The performance of the model in each subgroup was evaluated using the same metrics as those used for the overall model, including AUC, sensitivity, specificity, calibration curves, Brier score, and DCA.

### Statistics

Statistical analyses were conducted using R software (version 4.5.3; R Foundation). Measurement data with a normal distribution were expressed as means ± standard deviations (± s), and comparisons between groups were analyzed using the t-test. For continuous variables with a skewed distribution, data were presented as medians with interquartile ranges and compared using either the Mann-Whitney U test or the Kruskal-Wallis H test. Categorical variables were reported as counts and percentages and compared using the chi-square test. All continuous variables were z-normalized prior to inclusion in the logistic regression analysis to mitigate the effects of differences in variable units on the regression coefficients. Variance inflation factors were used to assess multicollinearity between variables before constructing the final multivariate logistic regression model. Variance inflation factors (VIFs) for all included variables were less than 5, indicating that collinearity issues were not problematic. During variable selection using LASSO regression, a ten-fold cross-validation (CV) procedure was employed to determine the penalty coefficient (λ). The final λ value was selected in accordance with the “one standard error (1-SE) rule” to obtain a more concise and robust model while maintaining predictive accuracy. The AUC was utilized to assess the predictive performance, and the optimal cut-off value was determined by the maximum Youden index (23). A p-value of less than 0.05 was deemed statistically significant.

## Results

### Clinical characteristics

**Table 1** summarizes the clinical characteristics of patients in the internal dataset. In the internal dataset, patients with HTN were older, were more likely to be male, and had a higher prevalence of DM than those without HTN. Laboratory findings showed that patients with HTN had significantly higher levels of TBIL, ALT, AST, BUN, SCr, Na, Mg, and AG. In contrast, RBC, ALB, and ALP levels were significantly lower in patients with HTN than in those without HTN.

**Table 1:** The comparison of the demographic and clinical characteristics of patients with hypothyroidism stratified by HTN status.

Variable	Non-HTN group (n = 1471)	HTN group (n = 1283)	P value
Age	53.00 (33.00–65.00)	70.00 (61.00–79.00)	<0.001
Gender	404 (27.46%)	588 (45.83%)	<0.001
DM	250 (17.00%)	545 (42.48%)	<0.001
WBC	6.70 (5.10–8.80)	6.50 (5.30–8.35)	0.934
RBC	4.10 (3.69–4.49)	4.06 (3.46–4.51)	0.006
Hb	123.00 (111.00–134.00)	123.00 (103.00–136.00)	0.081
Hct	0.37 (0.34–0.40)	0.37 (0.32–0.41)	0.063
MCV	91.50 (87.80–94.90)	91.60 (87.95–95.00)	0.573
MCH	30.43 (28.96–31.70)	30.34 (29.01–31.62)	0.794
MCHC	331.00 (323.00–339.00)	331.00 (321.00–339.00)	0.580
RDW	13.40 (12.60–14.60)	13.40 (12.70–14.80)	0.292
PLT	217.00 (175.00–268.00)	217.00 (174.25–266.75)	0.836
ALB	40.00 (36.00–43.00)	40.00 (35.00–44.00)	0.031
TBIL	8.10 (5.60–11.30)	8.50 (6.10–12.20)	0.005
ALT	16.00 (11.00–24.00)	18.00 (12.00–28.00)	<0.001
AST	20.00 (16.00–27.00)	21.00 (17.00–30.00)	<0.001
ALP	77.00 (58.38–112.00)	75.00 (60.00–96.00)	0.011
TG	1.42 (0.96–2.22)	1.37 (0.97–1.99)	0.110
BUN	4.50 (3.40–5.90)	6.20 (4.70–9.61)	<0.001
SCr	58.00 (47.00–73.00)	79.00 (62.00–116.00)	<0.001
K	4.10 (3.80–4.30)	4.10 (3.80–4.40)	0.411
Na	139.00 (137.00–142.00)	140.00 (137.00–143.00)	<0.001
Ca	2.27 (2.17–2.35)	2.25 (2.14–2.35)	0.067
Mg	0.85 (0.79–0.91)	0.88 (0.82–0.94)	<0.001
AG	16.50 (14.10–18.60)	17.50 (15.00–19.50)	<0.001

Continuous variables were compared using Student’s t-test or Mann–Whitney U test, and categorical variables were compared using χ² test. Statistical significance was defined as P <0.05. HTN, hypertension; DM, diabetes mellitus; WBC, white blood cell; RBC, red blood cell; Hb, hemoglobin; Hct, hematocrit; MCV, mean corpuscular volume; MCH, mean corpuscular hemoglobin content; MCHC, mean corpuscular hemoglobin concentration; RDW, red cell distribution width; PLT, platelet; ALB, albumin; TBil, total bilirubin; ALT, alanine aminotransferase; AST, aspartate aminotransferase; ALP, alkaline phosphatase; TG, triglycerides; BUN, blood urea nitrogen; SCr, serum creatinine; K, potassium; Na, sodium; Ca, calcium; Mg, magnesium; AG, anion gap.

### Variable selection

**Figure 1:**
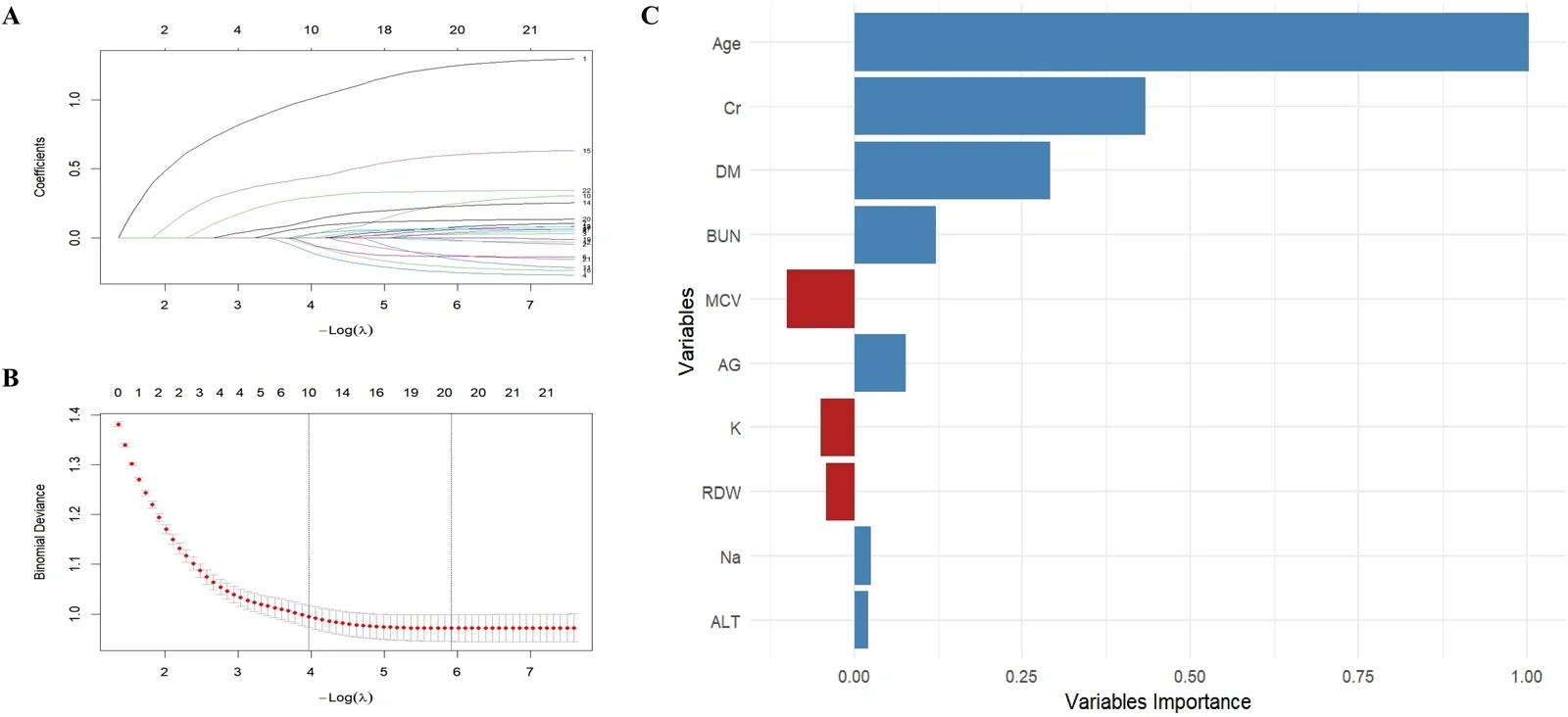
LASSO regression analysis was employed to identify the key variables associated with HTN among patients with hypothyroidism. **(A)** Path diagram of LASSO regression coefficients; **(B)** LASSO regression cross-validation curve; **(C)** Importance ranking diagram of LASSO variables. LASSO, least absolute shrinkage and selection operator; HTN, hypertension; SCr, serum creatinine; DM, diabetes mellitus; BUN, blood urea nitrogen; MCV, mean corpuscular volume; AG, anion gap; K, potassium; RDW, red cell distribution width; Na, sodium; ALT, alanine aminotransferase.

A total of 25 candidate variables were screened using LASSO regression, and 10 variables were identified as being significantly associated with HTN. The selected variables were age, SCr, DM, BUN, MCV, AG, K, RDW, Na, and ALT (**Figure 1**). To minimize the potential influence of multicollinearity, VIFs were subsequently calculated for these 10 variables. Three variables (MCV, K, and RDW) with VIFs >5 were excluded, leaving seven variables for subsequent logistic regression analyses.

### Collinearity assessment and logistic regression analysis

**Figure 2:**
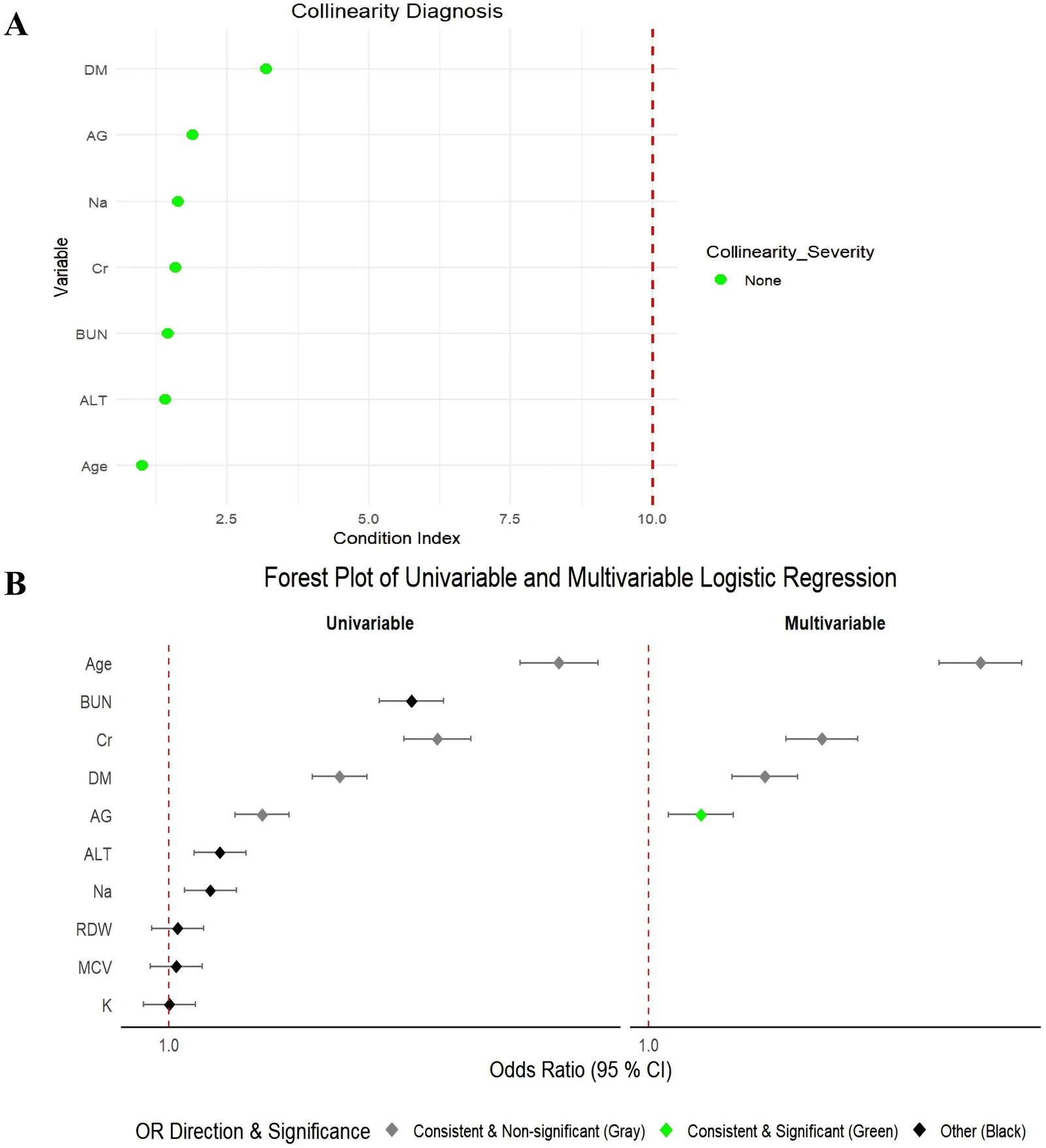
Collinearity diagnosis and regression analysis results of risk factors associated with HTN among patients with hypothyroidism. **(A)** Collinearity diagnosis of the independent variables. **(B)** Forest plots for univariate and multivariate logistic regression analysis. HTN, hypertension; BUN, blood urea nitrogen; SCr, serum creatinine; DM, diabetes mellitus; AG, anion gap; ALT, alanine aminotransferase; Na, sodium; RDW, red cell distribution width; MCV, mean corpuscular volume; K, potassium; OR, odds ratio.

Following LASSO-based variable selection, multicollinearity was assessed using VIFs. Three of the 10 LASSO-selected variables had VIFs > 5 and were excluded from subsequent analyses. The remaining seven variables, including DM, AG, Na, SCr, BUN, ALT, and age, all had VIFs < 5, indicating no substantial multicollinearity among the variables retained for multivariable logistic regression analysis (**Figure 2A**). Univariate logistic regression analysis showed that all seven variables were significantly associated with the risk of HTN (P < 0.05) (**Figure 2B**; **Supplementary Table 3**). Multivariate logistic regression analysis identified age (OR, 3.15; 95% CI, 2.73 - 3.64), SCr (OR, 1.82; 95% CI, 1.61 - 2.06), DM (OR, 1.49; 95% CI, 1.34 - 1.67) and AG (OR, 1.20; 95% CI, 1.07 to 1.34) as independent risk factors for HTN in patients with hypothyroidism (all P < 0.05). Comparison of the univariate and multivariate regression results showed that the directions of the associations for age, SCr, DM, and AG remained consistent, with no substantial attenuation or reversal of the effect estimates, indicating that these variables were robust and reliable predictors of HTN.

### Nomogram for predicting the risk of HTN in patients with hypothyroidism based on independent risk factors

**Figure 3:**
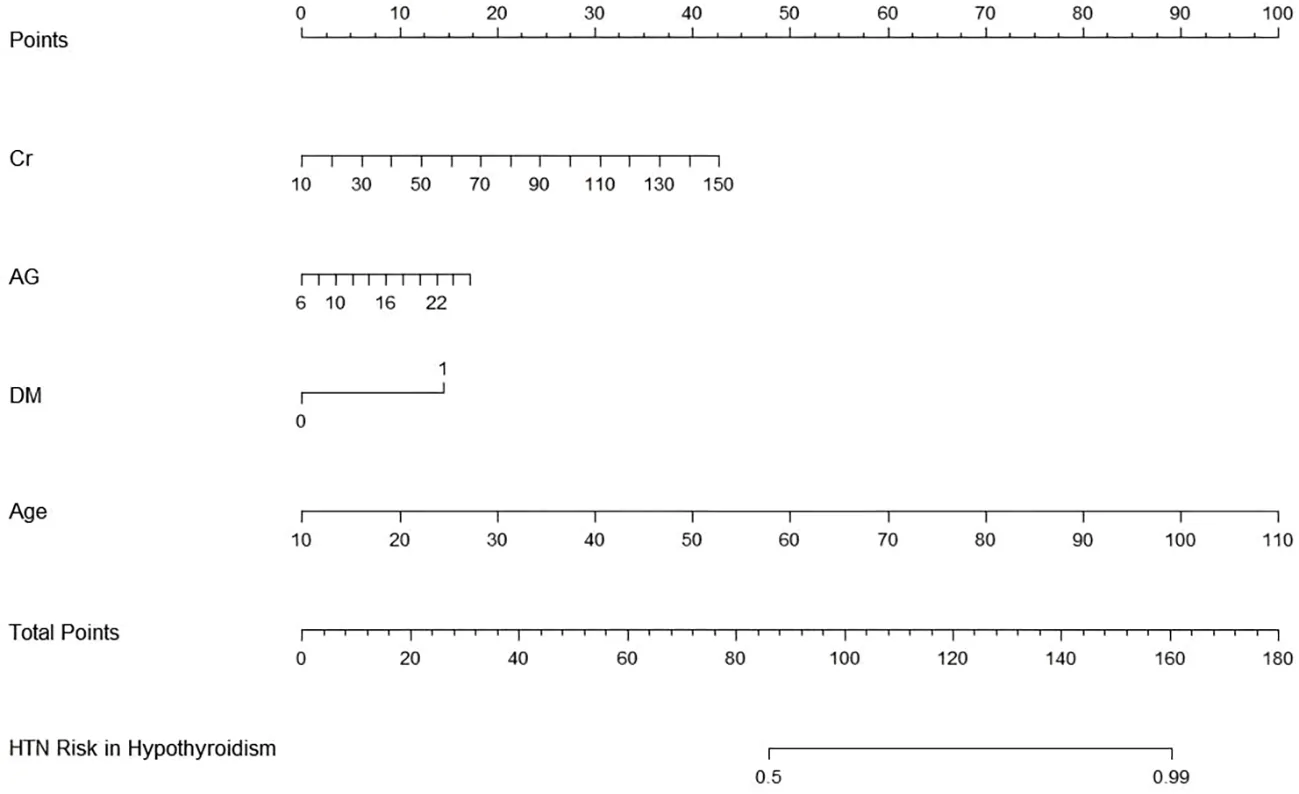
Nomogram model for predicting the risk of HTN among patients with hypothyroidism. HTN, hypertension; SCr, serum creatinine; AG, anion gap; DM, diabetes mellitus.

Based on the independent risk factors age, SCr, DM, and AG identified by multivariate logistic regression analysis, an individualized nomogram was developed to predict the risk of HTN (**Figure 3**). The regression coefficients of each variable were converted into corresponding point values, enabling quantitative assessment of individual risk factors. Age, SCr and AG were treated as continuous variables, with higher values corresponding to higher point scores. DM was treated as a binary variable, with the presence of DM assigned a fixed point value. The points assigned to each variable were summed to obtain the total score. The total score was then mapped to the corresponding probability of HTN in patients with hypothyroidism, allowing individualized risk estimation. A predicted probability below 0.5 was considered low risk, probabilities between 0.5 and 0.7 were categorized as moderate risk, and probabilities above 0.7 indicated high risk.

**Figure 4:**
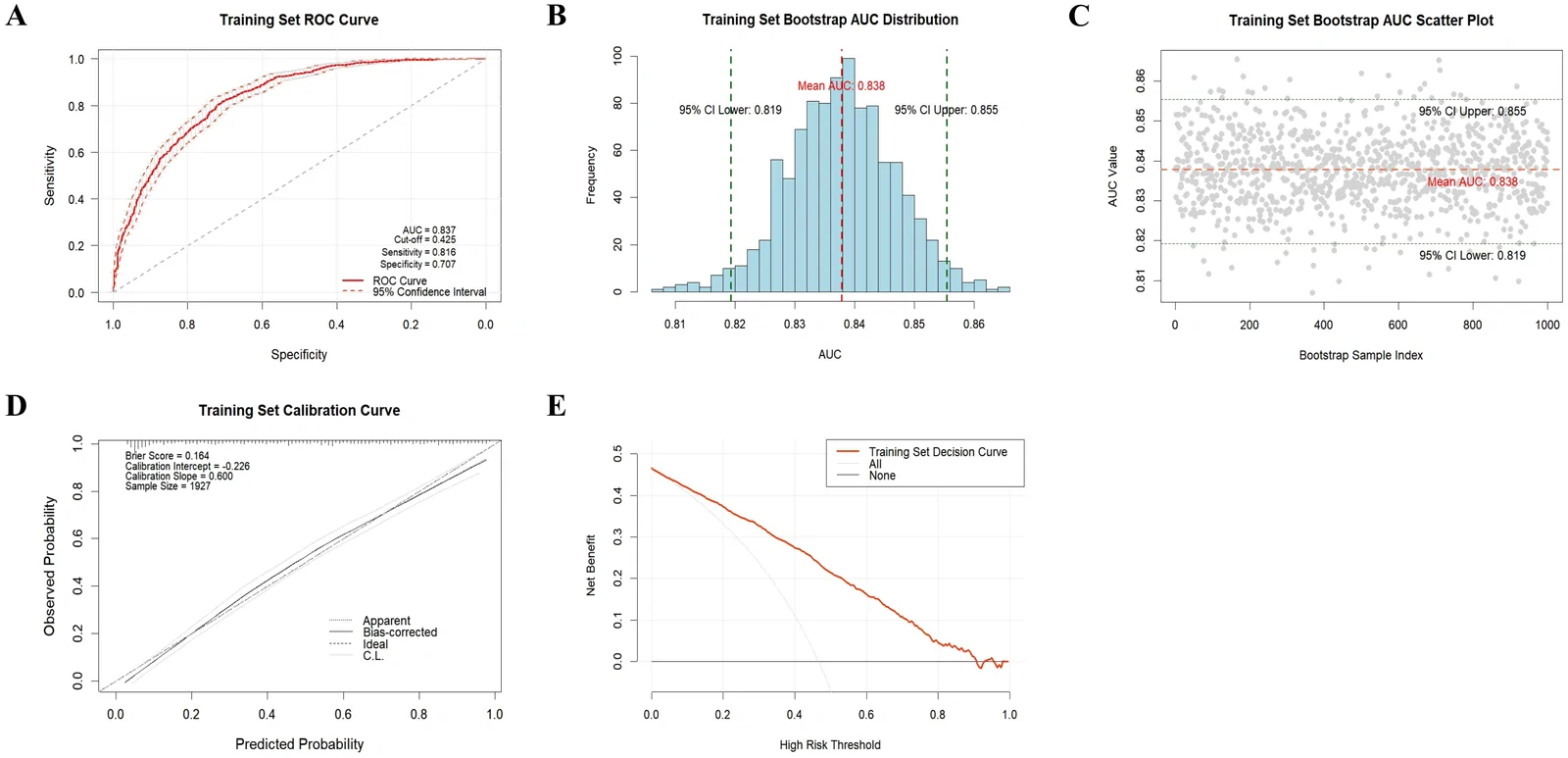
Comprehensive evaluation of the prediction nomogram model in the training set. **(A)** ROC curve of the training set; **(B, C)** AUC distribution and scatter plot based on 1,000 bootstrap repeated sampling; **(D)** Calibration curve of the training set; **(E)** DCA of the training set. ROC, Receiver operating characteristic; AUC, area under the curve; DCA, decision curve analysis.

The predictive performance of the nomogram was comprehensively evaluated in the training dataset. ROC curve analysis showed that the model achieved an AUC of 0.837, indicating good discriminative ability (**Figure 4A**). Bootstrap validation with 1000 resamples yielded an optimism adjusted AUC of 0.838 (95% CI, 0.807 - 0.846). The stable distribution of AUC values further confirmed the robustness of the model’s discriminative performance (**Figures 4B, C**). The calibration curve demonstrated good agreement between the predicted and observed probabilities, with a Brier score of 0.164 (**Figure 4D**). DCA demonstrated that the nomogram provided a greater net benefit than either the treat all or treat none strategy across most clinically relevant threshold probabilities, indicating good clinical utility (**Figure 4E**). Overall, the nomogram demonstrated good discrimination, calibration, and clinical utility in the training dataset.

### Generalization performance of the nomogram model in the test and external validation datasets

**Figure 5:**
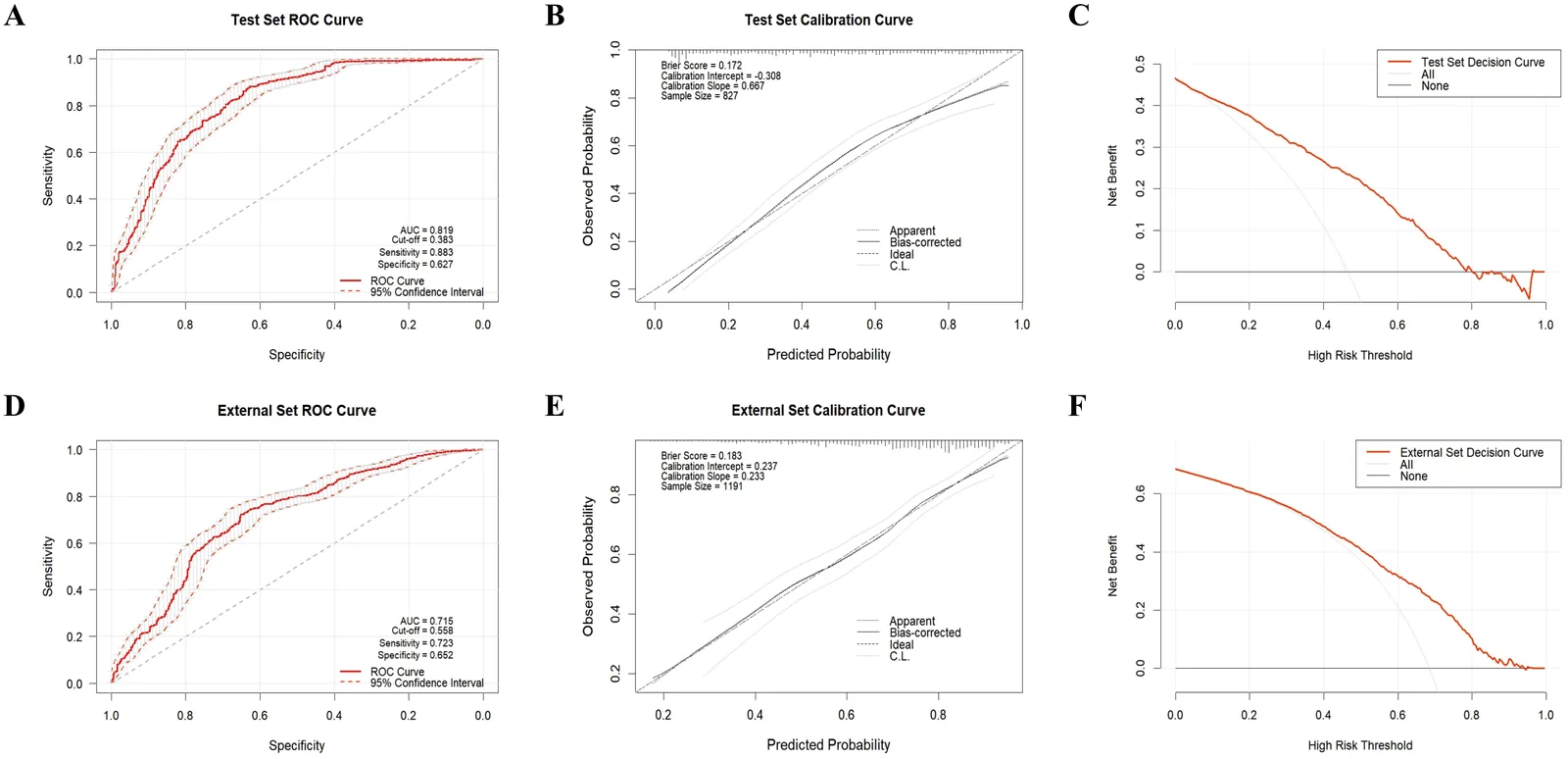
Evaluation of the generalization performance of the nomogram model in the test set and the external validation set. **(A)** ROC curve of the test set; **(B)** Calibration curve of the test set; **(C)** DCA of the test set; **(D)** ROC curve of the external validation set; **(E)** Calibration curve of the external validation set; **(F)** DCA of the external validation set. ROC, Receiver operating characteristic; DCA, decision curve analysis.

To evaluate the generalization performance of the model, independent validation was performed in the test dataset and the external validation dataset. In the test dataset, the model achieved an AUC of 0.819, demonstrating good discriminative ability (**Figure 5A**). The calibration curve demonstrated good agreement between the predicted and observed probabilities (Brier score = 0.172) (**Figure 5B**). DCA demonstrated that the model provided a greater net benefit than either the treat all or treat none strategy across a wide range of clinically relevant threshold probabilities, supporting its potential clinical usefulness. (**Figure 5C**).

In the external validation dataset, the model maintained good discriminative ability, with an AUC of 0.715 (**Figure 5D**). Calibration curve analysis demonstrated good agreement between the predicted and observed probabilities, with a Brier score of 0.183, supporting the model’s calibration in an independent population (**Figure 5E**). DCA further demonstrated that the model provided good net clinical benefit in the external validation cohort, supporting its generalizability across different populations (**Figure 5F**). Overall, the nomogram demonstrated stable discrimination, good calibration, and satisfactory clinical utility in both the test and external validation datasets, indicating good generalizability.

### Subgroup validation of the nomogram model

To examine whether the nomogram maintained its predictive performance across different patient groups, subgroup analyses were conducted according to gender. Among female participants, the model yielded an AUC of 0.863, whereas an AUC of 0.745 was observed in male participants, reflecting satisfactory discriminatory performance in both subgroups (**Supplementary Figures 1A, D**). Calibration assessment revealed close agreement between the estimated and observed risks, with Brier scores below 0.2 for both sexes, suggesting reliable probability estimation (**Supplementary Figures 1B, E**). Across clinically relevant threshold probabilities, DCA consistently favored use of the nomogram over the treat all and treat none strategies, supporting its potential value for clinical decision making (**Supplementary Figures 1C, F**).

Age specific analyses were subsequently performed to further assess model robustness. The nomogram achieved AUC values of 0.812 in participants younger than 60 years and 0.717 in those aged 60 years or older, indicating acceptable discrimination in both age groups (**Supplementary Figures 2A, D**). The corresponding calibration curves showed favorable agreement between predicted and observed event probabilities, while all Brier scores remained below 0.2, confirming stable calibration across age strata (**Supplementary Figures 2B, E**). Consistent with the findings from the gender based analyses, DCA showed that application of the nomogram resulted in a higher net benefit than either the treat all or treat none approach throughout the clinically relevant range of threshold probabilities, highlighting its potential clinical usefulness in both age groups (**Supplementary Figures 2C, F**).

## Discussion

In this multicohort study, we developed and externally validated a hypothyroidism-specific HTN risk prediction model using four routinely available clinical variables, including age, Cr, AG, and DM. The model demonstrated good discrimination, calibration, and clinical utility in the training cohort and maintained satisfactory performance in both internal testing and external validation cohorts. Furthermore, subgroup analyses showed consistent predictive ability across sex and age categories, supporting the robustness of the model. Notably, although 25 candidate variables were initially evaluated and 10 variables were selected through LASSO regression, only four factors remained independently associated with HTN after multivariable adjustment. These findings indicate that a limited set of clinically accessible indicators may capture key dimensions of HTN susceptibility among patients with hypothyroidism and provide a practical framework for individualized cardiovascular risk stratification.

To our knowledge, the primary contribution of our study is the first development and external validation of a disease specific HTN risk prediction model for patients with hypothyroidism. This approach addresses the unmet need for individualized risk stratification in a population with distinct endocrine and metabolic characteristics. Existing hypertension prediction models, including the Framingham-based risk equation, were primarily developed in general populations and rely mainly on traditional cardiovascular risk factors, such as age, sex, blood pressure, body mass index, smoking status, and family history (24). Although these models have demonstrated clinical utility, their applicability to patients with hypothyroidism remains uncertain because they do not incorporate endocrine-specific metabolic abnormalities. Furthermore, previous thyroid-related studies have mainly focused on associations between thyroid dysfunction and cardiovascular outcomes, such as coronary heart disease, heart failure, and mortality, rather than developing hypertension risk stratification tools specifically for individuals with hypothyroidism (25, 26). Consequently, a validated hypertension prediction model specifically developed for individuals with hypothyroidism remains unavailable. Although previous prediction models have often incorporated numerous demographic, biochemical, and lifestyle-related variables, excessive complexity may reduce interpretability and limit implementation in routine clinical practice (20). In contrast, our model achieved robust predictive performance using only four routinely available variables, including age, SCr, AG, and DM. These variables are routinely collected during clinical evaluation and require no additional specialized testing, thereby reducing implementation barriers and enhancing scalability across different healthcare settings. From a clinical perspective, the nomogram provides individualized risk estimation rather than relying solely on categorical classification of hypertension status. Patients identified as high risk may benefit from enhanced blood pressure monitoring, earlier lifestyle interventions, and comprehensive cardiovascular risk management. Furthermore, because all predictors are routinely available clinical variables, the model may be readily integrated into electronic health record systems or clinical decision support platforms to facilitate precision risk stratification among patients with hypothyroidism.

Age, SCr, AG, and DM included in the final model represent different but interconnected pathophysiological dimensions rather than a single biological pathway underlying HTN development in patients with hypothyroidism. Therefore, the model demonstrates biological plausibility. Thyroid hormone deficiency may reduce cardiac output, increase peripheral vascular resistance, and impair renal perfusion, thereby affecting glomerular filtration, sodium and water balance, and acid base homeostasis (27). Hypothyroidism is also frequently associated with insulin resistance, dyslipidemia, and chronic low grade inflammation, which may promote oxidative stress, endothelial dysfunction, and vascular remodeling, thereby increasing HTN risk (28, 29). Within this framework, age may reflect vascular aging and cumulative exposure to risk factors, whereas SCr may capture alterations in thyroid kidney axis function and volume regulation (30, 31). AG reflects disturbances in acid–base balance and systemic metabolic derangements, while DM represents persistent glucose dysregulation and related microvascular risk (32, 33). Previous studies have suggested that renal impairment, metabolic disturbances, and endothelial dysfunction associated with hypothyroidism may contribute to blood pressure elevation and cardiovascular remodeling. These four predictors should not be viewed as independent contributors to HTN risk. Rather, they may reflect interconnected biological alterations related to renal function, metabolic homeostasis, and vascular health in patients with hypothyroidism. As this is a clinical prediction model study, the above discussion aims to explain the biological plausibility of the identified predictors rather than establish causal mechanisms. These hypotheses require further evaluation through prospective and mechanistic studies.

The model demonstrated consistent predictive performance across the training, test, and external validation cohorts, supporting its potential clinical utility. Rather than relying solely on the AUC, this study incorporated calibration curves, Brier scores, and DCA to provide a comprehensive evaluation of model performance. Although AUC is widely used to assess discrimination, it does not evaluate calibration between predicted probabilities and observed risks or determine whether a model provides clinical benefit (34). Therefore, we evaluated model performance across three key domains: discrimination, calibration, and net clinical benefit. The results showed that the model achieved good discrimination, strong agreement between predicted and observed risks, and sustained net clinical benefit across a wide range of threshold probabilities, supporting the reliability of its predictive performance. The clinical applicability of this model is supported by its reliance on routinely collected clinical variables in routine practice. Unlike prediction tools that require specialized biomarkers or complex measurements, our nomogram incorporates four readily available predictors, which may facilitate implementation in routine endocrine practice. The model provides individualized risk estimation and may assist clinicians in identifying patients with hypothyroidism who may benefit from closer blood pressure monitoring and comprehensive cardiovascular risk assessment.

This study has several limitations that should be acknowledged. First, this retrospective observational study can identify associations between predictors and HTN development but cannot establish causal relationships. Second, disease specific variables, including thyroid-stimulating hormone, free thyroxine, thyroid autoantibodies, and thyroid hormone replacement therapy, were unavailable. Other potentially relevant factors, such as body mass index, lipid profiles, lifestyle behaviors, and medication use, were also not considered. Future studies may improve the model by incorporating thyroid function parameters, treatment information, and behavioral factors. Third, although external validation was performed, the generalizability of the model across different countries, ethnic groups, and healthcare systems requires further evaluation. In addition, this study developed a static prediction model based on baseline measurements, whereas thyroid function, renal function, and metabolic status may change over time. Future research may develop dynamic prediction models using longitudinal data, additional biomarkers, multi omics information, and artificial intelligence approaches to improve predictive performance over time. Prospective clinical impact studies are also needed to determine whether the model can improve clinical decision making and patient outcomes, thereby supporting its implementation in precision endocrinology practice.

## Conclusion

This study developed and validated a HTN risk prediction model for patients with hypothyroidism based on age, SCr, AG, and DM. The model demonstrated good predictive performance, clinical interpretability, external validity, and potential clinical utility. It may serve as an auxiliary tool for early risk identification and HTN risk stratification in patients with hypothyroidism. More importantly, our findings suggest that integrating routinely available clinical variables reflecting vascular aging, renal function, and metabolic homeostasis may improve characterization of cardiovascular susceptibility in patients with hypothyroidism. This approach may provide a basis for developing disease specific risk prediction models and advancing precision endocrinology.

## Data Availability

The data analyzed in this study is subject to the following licenses/restrictions: The data are available upon request from the authors. Requests to access these datasets should be directed to Jianbin You, 18344985224@163.com.
