# Supplementary material for: Development and external validation of a risk prediction model for hypertension among individuals with established hypothyroidism
Source: Front Endocrinol (Lausanne). 2026 Sep 3;17:1945343. doi: 10.3389/fendo.2026.1945343 (PMC13581942; doi:10.3389/fendo.2026.1945343)
Supplement: Supplementary file 1 [file SupplementaryFile1.docx]

**Supplementary Figures**

**Supplementary Figure 1. Evaluation of the generalization performance of the nomogram model in gender subgroups.** (A) ROC curve of the female subgroup; (B) Calibration curve of the female subgroup; (C) DCA of the female subgroup; (D) ROC curve of the male subgroup; (E) Calibration curve of the male subgroup; (F) DCA of the male subgroup. ROC: Receiver operating characteristic; DCA: decision curve analysis.

**Supplementary Figure 2. Evaluation of the generalization performance of the nomogram model in age subgroups.** (A) ROC curve of the < 60 years subgroup; (B) Calibration curve of the < 60 years subgroup; (C) DCA of the < 60 years subgroup; (D) ROC curve of the ≥ 60 years subgroup; (E) Calibration curve of the ≥ 60 years subgroup; (F) DCA of the ≥ 60 years subgroup. ROC: Receiver operating characteristic; DCA: decision curve analysis.

**Supplementary Tables**

**Supplementary Table 1. Disease codes included in the study.**

| **Diagnosis** | **ICD-9 codes** | **ICD-10 codes** |
| --- | --- | --- |
| Congenital hypothyroidism | 243 | E03.0, E03.1 |
| Postsurgical/Postprocedural hypothyroidism | 244.0, 244.1 | E89.0 |
| Iodine-related hypothyroidism | 244.2 | E02 |
| Drug-induced (iatrogenic) hypothyroidism | 244.3 | E03.2 |
| Postinfectious hypothyroidism | — | E03.3 |
| Acquired thyroid atrophy | — | E03.4 |
| Myxedema coma | — | E03.5 |
| Other specified hypothyroidism | 244.8 | E03.8 |
| Unspecified hypothyroidism | 244.9 | E03.9 |
| Essential hypertension | 401.0, 401.1, 401.9 | I10 |
| Hypertensive heart disease | 402.00–402.91 | I11.0, I11.9 |
| Hypertensive chronic kidney disease | 403.00–403.91 | I12.0, I12.9 |
| Hypertensive heart and chronic kidney disease | 404.00–404.93 | I13.0, I13.10, I13.11, I13.2 |
| Secondary hypertension | 405.01, 405.09, 405.11, 405.19, 405.91, 405.99 | I15.0, I15.1, I15.2, I15.8, I15.9 |
| Hypertensive crisis | — | I16.0, I16.1, I16.9 |
| **Diabetes mellitus** | **250.xx** | **E10.xx, E11.xx, E13.xx, E14.xx** |

ICD: international classification of diseases.

**Supplementary Table 2. Baseline characteristics of training and test sets.**

|  | **Training Set (n = 1927)** | **Test Set (n = 827)** | **P value** |
| --- | --- | --- | --- |
| DM | 545 (28.3%) | 250 (30.2%) | 0.323 |
| Gender | 679 (35.2%) | 313 (37.8%) | 0.206 |
| Age | 62.00 (26.00) | 62.00 (22.00) | 0.415 |
| AG | 16.90 (4.50) | 17.00 (4.45) | 0.203 |
| ALB | 40.00 (7.00) | 40.00 (9.00) | 0.939 |
| ALP | 76.00 (41.00) | 76.00 (42.30) | 0.812 |
| ALT | 17.00 (15.00) | 17.00 (15.00) | 0.285 |
| AST | 20.00 (12.00) | 21.00 (13.00) | 0.481 |
| BUN | 5.10 (3.20) | 5.30 (3.40) | 0.108 |
| Ca | 2.26 (0.19) | 2.27 (0.20) | 0.798 |
| SCr | 66.00 (35.00) | 67.00 (41.00) | 0.358 |
| Hb | 122.31 (25.00) | 123.00 (27.00) | 0.679 |
| Hct | 0.37 (0.07) | 0.37 (0.08) | 0.491 |
| K | 4.10 (0.60) | 4.10 (0.60) | 0.477 |
| MCH | 30.38 (2.59) | 30.36 (2.63) | 0.52 |
| MCHC | 331.00 (15.00) | 331.00 (16.00) | 0.646 |
| MCV | 91.50 (6.80) | 91.38 (7.00) | 0.417 |
| Mg | 0.87 (0.12) | 0.86 (0.13) | 0.248 |
| Na | 140.00 (5.00) | 140.00 (5.00) | 0.932 |
| PLT | 217.00 (89.00) | 219.00 (92.50) | 0.74 |
| RBC | 4.05 (0.86) | 4.07 (0.90) | 0.378 |
| RDW | 13.30 (1.90) | 13.50 (2.20) | 0.18 |
| TBIL | 8.20 (5.90) | 8.45 (5.75) | 0.652 |
| TG | 1.43 (1.14) | 1.47 (1.20) | 0.797 |
| WBC | 6.70 (3.30) | 6.60 (3.35) | 0.33 |

Continuous variables were compared using Student’s t-test or Mann–Whitney U test, and categorical variables were compared using χ² test. Statistical significance was defined as P <0.05. DM: diabetes mellitus; AG: anion gap; ALB: albumin; ALP: alkaline phosphatase; ALT: alanine aminotransferase; AST: aspartate aminotransferase; BUN: blood urea nitrogen; Ca: calcium; SCr: serum creatinine; Hb: hemoglobin; Hct: hematocrit; K: potassium; MCH: mean corpuscular hemoglobin content; MCHC: mean corpuscular hemoglobin concentration; MCV: mean corpuscular volume; Mg: magnesium; Na: sodium; PLT: platelet; RBC: red blood cell; RDW: red cell distribution width; TBil: total bilirubin; TG: triglycerides; WBC: white blood cell.

**Supplementary Table 3. Univariate and multivariate analyses of factors associated with HTN among patients with hypothyroidism.**

| **Variables** | **Univariate analysis** | | | | |  | **Multivariate analysis** | | | | |
| --- | --- | --- | --- | --- | --- | --- | --- | --- | --- | --- | --- |
|  | **β** | **SE** | **Z** | **P value** | **OR**  **(95% CI)** |  | **β** | **SE** | **Z** | **P value** | **OR**  **(95% CI)** |
| Age | 1.35 | 0.07 | 19.70 | < 0.001 | 3.85  (3.37 - 4.4) |  | 1.15 | 0.07 | 15.70 | < 0.001 | 3.15  (2.73 - 3.64) |
| MCV | 0.03 | 0.05 | 0.56 | 0.574 | 1.03  (0.94 - 1.12) |  |  |  |  |  |  |
| RDW | 0.03 | 0.05 | 0.67 | 0.5 | 1.03  (0.94 -1.13) |  |  |  |  |  |  |
| ALT | 0.18 | 0.05 | 3.85 | < 0.001 | 1.19  (1.09 - 1.31) |  |  |  |  |  |  |
| BUN | 0.84 | 0.06 | 14.80 | < 0.001 | 2.32  (2.07 - 2.59) |  |  |  |  |  |  |
| SCr | 0.93 | 0.06 | 15.70 | < 0.001 | 2.53  (2.25 - 2.84) |  | 0.60 | 0.06 | 9.48 | < 0.001 | 1.82  (1.61 - 2.06) |
| K | 0.00 | 0.05 | 0.05 | 0.958 | 1  (0.917 - 1.1) |  |  |  |  |  |  |
| Na | 0.14 | 0.05 | 3.12 | 0.002 | 1.15  (1.05 - 1.26) |  |  |  |  |  |  |
| AG | 0.32 | 0.05 | 6.80 | < 0.001 | 1.38  (1.26 - 1.52) |  | 0.18 | 0.06 | 3.17 | 0.002 | 1.2  (1.07 - 1.34) |
| DM | 0.59 | 0.05 | 12.10 | < 0.001 | 1.8  (1.64 - 1.99) |  | 0.40 | 0.06 | 7.00 | < 0.001 | 1.49  (1.34 - 1.67) |

HTN:hypertension; β: beta coefficient; SE: standard error; Z: Z - statistic; P: P value; OR: odds ratio; CI: confidence interval; MCV: mean corpuscular volume; RDW: red cell distribution width; ALT: alanine aminotransferase; BUN: blood urea nitrogen; SCr: serum creatinine; K: potassium; Na: sodium; AG: anion gap; DM: diabetes mellitus.

.
